# Using video-cases to assess student reflection: Development and validation of an instrument

**DOI:** 10.1186/1472-6920-12-22

**Published:** 2012-04-20

**Authors:** Sebastiaan Koole, Tim Dornan, Leen Aper, Bram De Wever, Albert Scherpbier, Martin Valcke, Janke Cohen-Schotanus, Anselme Derese

**Affiliations:** 1 Centre for Educational Development, Faculty of Medicine and Health Sciences, Ghent University, Ghent, Belgium; 2 Department of Educational Development and Research, Faculty of Health, Medicine and Life Sciences, Maastricht University, Maastricht, the Netherlands; 3 Department of Educational Studies, Faculty of Psychology and Educational Sciences, Ghent University, Ghent, Belgium; 4 Institute for Medical Education, Faculty of Health, Medicine and Life Sciences, Maastricht University, Maastricht, the Netherlands; 5 Centre for Research and Innovation in Medical Education, Faculty of Medical Sciences, University of Groningen and University Medical Centre Groningen, Groningen, the Netherlands

## Abstract

**Background:**

Reflection is a meta-cognitive process, characterized by: 1. Awareness of self and the situation; 2. Critical analysis and understanding of both self and the situation; 3. Development of new perspectives to inform future actions. Assessors can only access reflections indirectly through learners’ verbal and/or written expressions. Being privy to the situation that triggered reflection could place reflective materials into context. Video-cases make that possible and, coupled with a scoring rubric, offer a reliable way of assessing reflection.

**Methods:**

Fourth and fifth year undergraduate medical students were shown two interactive video-cases and asked to reflect on this experience, guided by six standard questions. The quality of students’ reflections were scored using a specially developed Student Assessment of Reflection Scoring rubric (StARS®). Reflection scores were analyzed concerning interrater reliability and ability to discriminate between students. Further, the intra-rater reliability and case specificity were estimated by means of a generalizability study with rating and case scenario as facets.

**Results:**

Reflection scores of 270 students ranged widely and interrater reliability was acceptable (Krippendorff’s alpha = 0.88). The generalizability study suggested 3 or 4 cases were needed to obtain reliable ratings from 4th year students and ≥ 6 cases from 5th year students.

**Conclusion:**

Use of StARS® to assess student reflections triggered by standardized video-cases had acceptable discriminative ability and reliability. We offer this practical method for assessing reflection summatively, and providing formative feedback in training situations.

## Background

The traditional view that learning results from transmission of knowledge is shifting towards a view that actively constructed knowledge underpins self-regulated and lifelong learning [1,2]. The concept of meta-cognition - awareness and active control over cognitive processes - is central to self-regulated learning [3-5]. Reflection is an essential part of meta-cognition. It is conceived of as a cyclic process comprising monitoring, evaluating, and planning [3,6]. Boud et al. [7] defined reflection as “a generic term for those intellectual and affective activities in which individuals engage to explore their experiences in order to lead to a new understanding and appreciation” (p.19). In line with this definition, three elements of reflection have been identified: 1. Awareness of self and the situation; 2. Critical analysis and understanding of both self and the situation; 3. Development of new perspectives to inform future actions [7-10].

Schön’s concept of the ‘Reflective Practitioner [11,12] captured the central place of reflection in professional practice. He identified it as a means of revisiting personal experience to learn and manage complex problems encountered in professional contexts. In health care sciences, the ability to reflect on experiences is regarded as an important attribute that allows professionals to respond to the demands of the complex environments they work in [13-15]. It helps them identify shortcomings in their knowledge and skills, and understand their professional actions better [16,17]. Accordingly, many policy documents have identified reflection on professional experiences as an important outcome parameter for graduated physicians [18-20]. There is, however, a discrepancy between the growing consensus that reflection on professional experience is beneficial and the persisting lack of clarity about the best methods to teach and assess it [9,21]. Education and assessment are interrelated. Assessment is needed to measure whether learners have achieved required learning goals, indirectly identifying the efficiency of the used educational method. It can also impact directly on learning by providing feedback on strengths and weaknesses that allows students to control and structure their learning [22,23].

The fact that reflection is a meta-cognitive process complicates assessment because it implies a process of thought only accessible to the reflecting person [7,9]. Assessors can only observe this process indirectly through verbal and/or written expressions. Moreover, they usually access reflective thoughts without any knowledge of the situation that stimulated them. To put reflective thought into its proper context, it would be valuable if assessors had access to the triggering situation as well as the thought it provoked. In order to access the triggering situation assessors could be asked to observe situations live or by video but the time involved would make assessment of whole cohorts of learners impractical. As an alternative, Hulsman et al. [24] asked students to review video recordings of their performances and select key fragments in which to ground their written reflections. Students had also to review video recordings of other students and provide peer feedback. This self and peer orientated approach solved the time efficiency issue, but presented only a selected and fragmented window into the triggering situation and depended on peers understanding reflection well enough to provide valuable feedback.

Vignettes or short stories based on simulations of real events can be used to stimulate reflection [25]. Boenink et al. [26] demonstrated the utility of paper vignettes to assess student reflections. Balslev et al. [27] and Kamin et al. [28] found that video-cases triggered critical thinking better than written cases. Similar results were found by Botezatu et al. [29], who used virtual patient simulation for both education and assessment. In the context of communication training in the third year of an undergraduate medical curriculum, Hulsman et al. [30] found that short questions about standardized video-cases concerning history taking, breaking bad news and decision making could ground reliable and discriminating scoring. Also in the domain of communication skills, Mazor et al. [31] showed that video-vignettes could provide good generalizability estimates. These findings suggest the use of such standardized video-cases to trigger reflection for the purpose of assessment as a worthy approach for further study.

To score written reflections various coding schemes have been proposed, using from three to seven categories [32,33] and introducing a variety of indicators [34]. Wong et al. [32] showed there was a tension between the reliability of coding schemes and their ability to discriminate between learners; a smaller number of categories had acceptable reliability but limited ability to discriminate whilst a larger number was more discriminant but less reliable. Recently, scoring rubrics have been used to score reflections [35-37]. These are scoring guides, which provide quality definitions that enable assessors to score efficiently and support learning in a way that can contribute to instructional quality [38,39]. Building on the reported findings about standardized video-cases and scoring rubrics, the current study replaced live situations with video-cases to trigger reflection within a standardized context. A scoring rubric was developed to score reflection reliably. Our objectives, then, were to:

1. Pilot an assessment method combining standardized video-cases to stimulate student reflection on consultation experiences and a scoring rubric to measure it, which could be used for training and to provide feedback.

2. Evaluate reflection scores resulting from this method in terms of:

their ability to discriminate between students

their reliability, as judged by inter-rater and intra-rater variation, and case-specificity

## Methods

### Development of video-cases to trigger student reflections

To trigger reflections, we developed four interactive video-cases, recorded from a physician’s perspective to increase their authenticity. Scripts were drafted by skills lab teachers and patient roles were played by experienced simulated patients who had received five hours of training. Each video-case showed a patient consulting a general practitioner with a problem appropriate to students’ expected level of competence. All cases followed the same structure: reason for encounter, history, physical examination, explanation of diagnosis, advice and treatment planning, and closure of the consultation. Each case lasted 15–20 minutes, similar to real life consultations.

**Figure 1:**
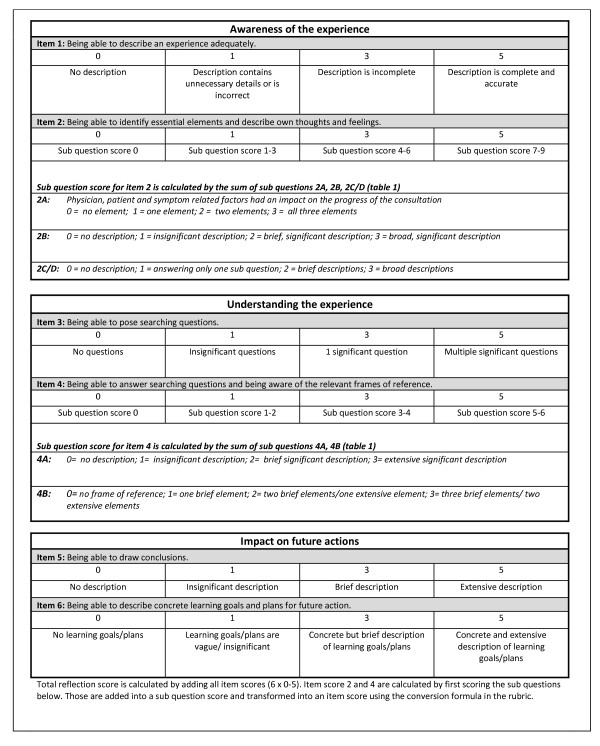
Student Assessment of Reflection Scoring rubric (StARS®) used to calculate an overall reflection score.

The video-cases were made interactive to stimulate student involvement. The interactive element consisted of six interruptions. At each interruption the screen turned black and a question appeared, like “How would you react now?” or “What diagnosis do you think is appropriate and why?“. The questions were formulated to confront students with complex and multidimensional problems that could not be solved in a straightforward way in order to stimulate reflection [11,12]. While students were writing down their answers, a countdown timer informed them when the video-case would resume. The time limit was introduced to make the video-cases like real consultations where there is only limited time to think. Having finishing a video-case, students were asked to reflect on their experience. Whilst reflection is characterized by a number of key elements, the boundaries between them are often blurred in reality [7,40]. People seldom take every step in full awareness and in strict succession. It is difficult to compare such diverse reflections. Hence we introduced six questions (Table 1) to structure student reflections. These questions were developed to represent the three key elements of reflection (2 questions/element) as described in the ‘introduction’ (awareness, understanding and future actions). Afterwards these structured reflections were scored using the Student Assessment of Reflection Scoring rubric (StARS®) (Figure 1).

**Table 1:** Reflection structuring questions posed after the interactive video-case to guide students through the process of reflection

***Aspect of reflection process***	***Question***
*Awareness of the experience*	*1. Describe the progress of the consultation with attention to both patient behavior and the physician’s actions.*
	*2. A What people or factors had an impact on the progress of the consultation?* B What did you think/feel when answering the case question?* C Looking back on the progress of the consultation: what went well? *D What did not go well?*
*Understanding the experience*	*3. Formulate searching questions that help to analyze your own actions/thoughts during the consultation process.*
	*4. A Try to answer your searching questions.* *B What knowledge/feelings/values/former experiences did you use to formulate your answer(s)?*
*Impact on future actions*	*5. What did you learn going through this consultation?*
	*6. What concrete actions did you plan for future practice?*

* In each case a question was selected that put students in a stressful and acute situation that demanded a reaction.

### Development of a rubric to assess student reflections

The StARS® is based on a scoring grid developed by Duke and Appleton [34] retaining only the items related to the construct of reflection. This resulted in a 5-item scoring rubric, which we complemented with an item about searching questions to represent the construct of reflection fully [10,41]. Item descriptions of the scoring rubric were tested for ambiguity in a pilot study among sixth year undergraduate medical students at Ghent University. After a consultation exercise with a simulated patient, four students were asked to reflect on this experience guided by the reflection structuring questions. Their structured reflections were independently scored by three assessors (SK, LA and AD) using the scoring rubric. Afterwards item descriptions displayed in the rubric were discussed by the assessors and, when experienced as unclear, revised accordingly. The number of scoring options was also reduced and boundaries between them were clarified, to minimize inconsistency between assessors. After revision, StARS® consisted of 6 items (2 items/element), to be scored on a 4-point scale. A total absence of any reflective expression in a scoring item is identified by 0. Because the presence of insignificant expressions are closer to no expressions than to significant expressions, 0, 1, 3, 5 scale was used. The 6 score items together are added to provide an overall reflection score (range 0–30). Good reflection, according to StARS® is:

A comprehensive and accurate view of an experience with attention to one’s own and others’ thoughts and feelings and an ability to make a distinction between essential and less important facets of the experience.

Being able to explore the experience with searching questions and being aware of the frames of reference used to answer those questions.

Being able to draw conclusions and translate them into concrete action plans for future practice.

### Participants and procedures

This study was approved by the ethical committee of Ghent University Hospital. In the academic year 2008–2009, all fourth (n = 206) and fifth year (n = 156) undergraduate medical students at Ghent University were invited to participate. Those who accepted had to attend two sessions in which they completed an interactive video-case and reflected on their experiences of the case. Each student completed two different cases in the same order, the content of which was related to the curriculum modules of the previous semester. Fourth year cases were about ventricular fibrillation (C1) and heart failure (C2); fifth year cases were about transient ischemic attack (C3) and neck/arm pain (C4). To limit interaction bias, all sessions using the same video-cases were held successively on a single day.

Student wrote their answers to the guiding questions on paper forms, which were scored with StARS®. All student reflections were scored by the same assessor (SK).

### Analysis

As we intended this method to be used by skills lab teachers, we recruited two teachers who were experienced in skills lab consultation training, but had neither been trained in marking reflective writings, nor involved in the development of StARS®. They were asked to score 40 randomly selected student reflections.Their training consisted of a 30 minute introductory session in which the underlying concept of reflection and the rubric were explained and they scored one student reflection to be discussed together afterwards. They then independently scored student reflections, from which we calculated the inter-rater variance using Krippendorff’s alpha (Kalpha). Hayes and Krippendorff [42] reported that many commonly used reliability coefficients such as Scott’s pi, Cohen’s kappa, and Cronbach’s alpha are either limited to two observers, fail to control for chance agreement, or only use corrections for the number of categories and not the distribution of ratings across categories or intervals. In order to overcome these limitations, they proposed Kalpha, useable for any number of raters, level of measurement, and sample size, accommodating missing data and controlling for chance agreement.

In addition, all student reflections were scored by one assessor (SK) and results were analyzed by descriptive statistics (mean, standard deviation and range) to explore the method’s ability to discriminate between students.

Intra-rater variance was investigated by the same assessor (SK) scoring all student reflections for a second time 18 months apart. These data resulted in 4 reflection scores for each student (2 cases with each being scored twice), which were used in a generalizability study to analyze intra-rater and case specificity as possible sources of variance in reflection scores. A generalizability study shows the relative size of each source of variation and their interactions, which together provide a generalizability coefficient (G coefficient) between 0 and 1. This measure indicates whether differences observed between students are real. G values of 0.8 and higher are generally accepted as a threshold for high-stake judgments [43]. To investigate how the reliability of reflection scores could be optimized, G coefficients were calculated, varying number of cases and ratings in a decision or D study. All statistical analyses were performed using SPSS 17.0 (SPSS Inc., Chicago, IL, USA). To calculate the Kalpha a macro downloaded from http://www.afhayes.com/spss-sas-and-mplus-macros-and-code.html was used in SPSS. G- and D studies were performed with a macro for SPSS downloaded from https://people.ok.ubc.ca/brioconn/gtheory/gtheory.html.

## Results

181 fourth year (88%) and 92 fifth year students (59%) reflected on two cases (C1 and C2 for fourth year students, C3 and C4 for fifth year students) and could therefore be included in the statistical analysis. Non-participation was due to circumstances like timetable clashes and illness, which were unlikely to have systematic effects on the findings.

Individual students’ reflection scores ranged between 1–30 with a mean overall reflection score of 19.1 (SD 4.5) as shown in Table 2. A Kalpha coefficient of 0.88 demonstrated acceptable inter-rater reliability between the scores of the two skills lab teachers. The variance components of generalizability studies in a two-facet crossed design with rating and case as facets performed separately for fourth and fifth year students to limit student variation are detailed in Table 3. The D study, shown in Table 4, indicated that G coefficients of reflection scores could be improved by increasing the number of cases while increasing the number of ratings by the same rater had no substantial effect.

**Table 2:** Student Assessment of Reflection Scoring rubric (StARS®) used to calculate an overall reflection score

**Case**		**Reflection score**			
		**Mean**	**SD**	**Range**	**Total of student**
4th year	C1	20.1	4.3	7-30	181
	C2	17.6	4.7	1-26	181
5th year	C3	20.2	4.2	8-30	92
	C4	19.08	4.0	8-28	92

Each item is scored on a scale of 0-5.

**Table 3:** Contributions of student, rating, and case and their interactions as sources of variance (variance estimate VE and relative contribution RC) in reflection scores

**Component**	**Fourth year students**		**Fifth year students**	
	**VE**	**RC**	**VE**	**RC**
Student	11.11	0.39	5.51	0.34
Rating	0.00	0.00	0.00	0.00
Case	5.17	0.20	0.05	0.00
Student x Rating	0.00	0.00	0.75	0.05
Student x Case	6.90	0.26	6.83	0.43
Case x Rating	1.02	0.04	0.26	0.02
Student x Case x Rating	2.92	0.11	2.60	0.16
**G coefficient**	** 0.71**		** 0.55**	

**Table 4:** D study to investigate the effect of more ratings by the same assessor and more cases on the G coefficients in fourth and fifth year student reflection scores

**Cases**	**Fourth year students**				**Fifth year students**			
	**1 rating**	**2 ratings**	**3 ratings**	**4 ratings**	**1 rating**	**2 ratings**	**3 ratings**	**4 ratings**
1	0.51	0.55	0.56	0.57	0.35	0.39	0.41	0.42
2	0.67	0.71	0.72	0.73	0.50	0.55	0.57	0.58
3	0.76	0.78	0.79	0.80*	0.59	0.64	0.66	0.67
4	0.81*	0.83*	0.84*	0.84*	0.64	0.70	0.72	0.73
5	0.84*	0.86*	0.87*	0.87*	0.68	0.73	0.76	0.77
6	0.86*	0.88*	0.89*	0.89*	0.70	0.76	0.78	0.80*
7	0.88*	0.89*	0.90*	0.90*	0.72	0.78	0.80*	0.81*
8	0.89*	0.91*	0.91*	0.91*	0.74	0.80*	0.82*	0.83*
9	0.90*	0.92*	0.92*	0.92*	0.75	0.81*	0.83*	0.84*
10	0.91*	0.92*	0.93*	0.93*	0.77	0.82*	0.84*	0.86*

* identifies an adequate number of cases and ratings to achieve a G coefficient ≥ 0.80.

Descriptive statistics (Table 2) have indicated a wide variation in reflection scores (range and standard deviation), which suggest the used method can discriminate between students. An alternative explanation, that inaccurate measurement could cause these wide ranged scores, proved inconsistent with the measured inter-rater and intra-rater reliability, that were satisfactory. Together, these findings provide evidence in support of a valid measure of inter-individual differences in reflection.

## Discussion

We have developed a method of assessing student reflections using standardized video cases and a scoring rubric, applied it to 270 fourth and fifth year undergraduate medical students, and demonstrated that the resulting reflection scores have acceptable psychometric properties including the ability to discriminate, inter- and intra-rater reliability, and case-specificity.

Replacing situations unique to individual students with standardized video-cases provided a common base for assessment without limiting variance between reflection scores. This variance can be attributed to two factors. First, students have unique frames of reference influenced by their individual prior experiences, knowledge, and beliefs [44], which lead them to reflect on different aspects of experience, pose different searching questions, and identify different learning goals. Second, the scoring items of StARS® identify the process of reflection (eg. the ability to ask searching questions or to draw conclusions) and this process varies independently of the content of reflection which is related to the triggering situation [41].

The inter-rater reliability of skills lab physicians, who had been trained for only 30 minutes, was sufficient. This finding reflects favourably on the use of guiding questions to structure reflections and the quality of the scoring rubrics. Each rater took about three hours to score 40 student reflections, proving StARS® is a practical instrument to evaluate student reflections in order to provide feedback.

Feedback about reflection is becoming increasingly important as the idea of reflection as a strictly individual internal process is changing into a notion of a thinking process that needs to be complemented with external feedback. This increased focus on external information is grounded in concerns about individuals lacking accurate introspection skills to fuel reflections and recognition of a need to verify one’s reflecting thoughts and frame of reference against a broader perspective [45]. Discussing experiences and the reflective thoughts that accompany them is key to bringing an internal process and external information together. Multiple formats have been proposed such as critical friends, formative feedback from supervisors and peer feedback [46-48]. However, interacting effectively about reflections, requires individuals to learn to verbalize their reflective thoughts. Our proposed method of assessment through facilitated reflection may be beneficial for this learning process as it structures reflections by means of structuring questions and provides feedback on essential aspects of the process of reflection as StARS® items are scored.

The generalizability study identified students, cases, and the interaction between them to be the main sources of variance in reflection scores. The variability between students is evidence of systematic individual differences in the quality of reflection and is not to be seen as error [49]. Variance between cases (case specificity), however, was an important source of error. The D study showed that increasing the number of cases had a much greater effect on the G coefficient than increasing the number of ratings. The content of cases and reflections that ensue from them have a complicated relation. According to Schön [11,12] a complex, challenging context best stimulates reflection. We tried to match video-cases to students’ expected level of competence but it is likely individual students found different levels of challenge in the same cases and were therefore stimulated differently by them. As well as case-related effects, Kreiter and Bergus [50] recommended considering occasional influences like momentary insights and confusions as possible confounders. Despite those considerations, three to four cases (depending on the number of ratings) were enough to obtain the G coefficient of 0.80 needed for high stakes decisions in fourth year students, though fifth year students needed over six cases [43]. This result suggests the usage of this method spread over time during a course rather than on one day high stakes exams as students need approximately 1 hour to view a case and to reflect upon.

Whilst the standardized context of video-cases is useful for training and assessment purposes, it also introduces a limitation. The ultimate aim of reflection is to learn from experiences so future actions can be more purposeful and deliberate [16]. In real life, students choose which experiences to reflect on, related to their individual development as physicians-to-be and life-long learners. Fueled, as they are, by less personal and meaningful experiences, reflections based on standardized video-cases might have a lesser impact on individual learning. That disadvantage may, however, be offset by the advantages of giving feedback on reflection that is informed by detailed knowledge of the triggering situation.

It could be argued that using a 4-point scale in StARS® (0,1,3,5) limits the diversity of reflection scores and hence discrimination between students. Our findings do not, however, support that claim as scores ranged between 0–30 with standard deviations above 4.0 in each year and for each case. Reflection scores were calculated as the sum of the scores on the 6 items in the rubric. That had the benefit of showing differences in students’ overall ability to reflect but could also hide important differences between students with similar total scores. Totally different patterns of item scores, resulting from students’ diverse reflection strategies could result in similar aggregate scores .

It could be questioned whether the 6-item structure of StARS® adequately represents the process of reflection. In fact, we reviewed the literature very carefully to search for items that were common to the various widely-used models/theories of reflection to develop the scoring rubric [10]. Use of those common items to construct StARS® is an important factor contributing to its validity.

Medical students have a constant stream of encounters with colleagues, supervisors, patients, their families, and other health care workers. This continuous series of interrelated events, and the reflections they trigger are wide open to further research. The aim of the present study was to develop a method of meeting this complex educational challenge under well-defined, standardized lab conditions. Comparison with the learners’ ability to reflect in more complex and authentic situations in real life is the next challenge. Further research, however will have to identify how to standardize the stimulus for these authentic reflections and how to make it possible for an assessing third party to observe them in whole populations of students. Furthermore, future research could focus on the relation between acquired reflection scores and academic or medical performance since empirical evidence about the effects of reflection on practice remain scarce [21].

## Conclusion

Reflections triggered by standardized video-cases and assessed with StARS® could be scored with acceptable discrimination between students, inter-rater reliability and generalizability properties concerning intra-rater and case specificity. We offer this practical method for assessing reflection summatively, and providing formative feedback in training situations.

## Competing interests

The authors declare that they have no competing interests.

## Authors’ contributions

SK, AD and MV conceptualized the idea, SK, LA and AD conducted the investigation, SK and BDW performed the statistical analysis and SK, TD and MV were involved in writing the initial drafts. All authors were involved in the revised drafts and made essential contributions to this paper and critically reviewed and approved the final manuscript.

## Pre-publication history

The pre-publication history for this paper can be accessed here:

http://www.biomedcentral.com/1472-6920/12/22/prepub

## References

[B1] Cornford IR Learning-to-learn strategies as a basis for effective lifelong learning International Journal of Lifelong Education 2002 21 4 357 368 10.1080/02601370210141020

[B2] Sperling RA Howard BC Staley R DuBois N Metacognition and Self-Regulated Learning Constructs Educ Res Eval 2004 10 2 117 139 10.1076/edre.10.2.117.27905

[B3] Brown AL Weinert FEKRH Metacognition, executive control, self-regulation and other more mysterious mechanisms Metacognition, motivation and understanding 1987 Laurence Erlbaum Associates, Hillsdale, New Jersey 65 116

[B4] Moos D Azevedo R Self-efficacy and prior domain knowledge: to what extent does monitoring mediate their relationship with hypermedia learning? Metacognition and Learning 2009 4 3 197 216 10.1007/s11409-009-9045-5

[B5] Schraw G Dennison RS Assessing Metacognitive Awareness Contemp Educ Psychol 1994 19 4 460 475 10.1006/ceps.1994.1033

[B6] Schraw G Promoting general metacognitive awareness Instr Sci 1998 26 1–2 113 125

[B7] Boud D Keogh R Walker D Reflection Turning experience into learning 1985 Kogan Page, London

[B8] Atkins S Murphy K Reflection - A Review of the Literature J Adv Nurs 1993 18 8 1188 1192 10.1046/j.1365-2648.1993.18081188.x 8376656

[B9] Sandars J The use of reflection in medical education: AMEE Guide No. 44 Medical Teacher 2009 31 8 685 695 10.1080/01421590903050374 19811204

[B10] Koole S Dornan T Aper L Scherpbier A Valcke M Cohen-Schotanus J Derese A Factors confounding the assessment of reflection: a critical review BMC Medical Education 2011 11 104 10.1186/1472-6920-11-104 22204704 PMC3268719

[B11] Schön DA Educating the reflective practitioner 1987 Jossey-Bass, San Fransisco

[B12] Schön DA The reflective practitioner: How professionals think in action 1983 Basic Books, New York

[B13] Plack MM Greenberg L The Reflective Practitioner: Reaching for Excellence in Practice Pediatrics 2005 116 6 1546 1552 10.1542/peds.2005-0209 16322184

[B14] Robertson K Reflection in professional practice and education Aust Fam Physician 2005 34 9 781 783 16184213

[B15] Kjaer NK Maagaard R Wied S Using an online portfolio in postgraduate training Medical Teacher 2006 28 8 708 712 10.1080/01421590601047672 17594582

[B16] Bethune C Brown JB Residents' use of case-based reflection exercises Can Fam Physician 2007 53 3 470 476 PMC1949082 17872683

[B17] Branch WTJ Paranjape AM Feedback and Reflection: Teaching Methods for Clinical Settings Academic Medicine 2002 77 12 1185 1188 10.1097/00001888-200212000-00005 12480619

[B18] General medical council Tommorow's Doctors 2009 GMC, London http://www.gmc-uk.org/TomorrowsDoctors_2009.pdf_27494211.pdf [accessed October 28th 2010]

[B19] Van Herwaarden CLA Laan RFJM Leunissen RRM The 2009 framework for undergraduate medical education in the Netherlands 2009 Dutch Federation of University Medical Centres, Utrecht http://www.nfu.nl/fileadmin/documents/Raamplan2009engelstalige_versie.pdf [accessed January 17th 2011].

[B20] Scottish Deans’ Medical Curriculum Group Learning Outcomes for the Medical Undergraduate in Scotland A Foundation for Competent and Reflective Practitioners 2007 3 rd http://www.scottishdoctor.org 10.1080/01421590220120713 12098432

[B21] Mann K Gordon J MacLeod A Reflection and reflective practice in health professions education: a systematic review Adv Heal Sci Educ 2009 14 4 595 621 10.1007/s10459-007-9090-2 18034364

[B22] Tillema HH Assessment for Learning to Teach Appraisal of Practice Teaching Lessons by Mentors, Supervisors, and Student Teachers J Teach Educ 2009 60 2 155 167 10.1177/0022487108330551

[B23] Cilliers FJ Schuwirth LW Adendorff HJ Herman N van der Vleuten CP The mechanism of impact of summative assessment on medical students' learning Adv Heal Sci Educ 2010 15 5 695 715 10.1007/s10459-010-9232-9 PMC2995206 20455078

[B24] Hulsman RL Harmsen AB Fabriek M Reflective teaching of medical communication skills with DiViDU: Assessing the level of student reflection on recorded consultations with simulated patients Patient Education and Counseling 2009 74 2 142 149 10.1016/j.pec.2008.10.009 19062232

[B25] Spalding NJ Phillips T Exploring the use of vignettes: From validity to trustworthiness Qual Heal Res 2007 17 7 954 962 10.1177/1049732307306187 17724107

[B26] Boenink AD Oderwald AK de Jonge P van Tilburg W Smal JA Assessing student reflection in medical practice. The development of an observer-rated instrument: reliability, validity and initial experiences Medical Education 2004 38 4 368 377 10.1046/j.1365-2923.2004.01787.x 15025638

[B27] Balslev T De Grave WS Muijtjens AMM Scherpbier AJJA Comparison of text and video cases in a postgraduate problem-based learning format Medical Education 2005 39 11 1086 1092 10.1111/j.1365-2929.2005.02314.x 16262803

[B28] Kamin C O'Sullivan P Deterding R Younger M A comparison of critical thinking in groups of third-year medical students in text, video, and virtual PBL case modalities Academic Medicine 2003 78 2 204 211 10.1097/00001888-200302000-00018 12584102

[B29] Botezatu M Hult H Tessma MK Fors UGH Virtual patient simulation for learning and assessment: Superior results in comparison with regular course exams Medical Teacher 2010 32 10 845 850 10.3109/01421591003695287 20854161

[B30] Hulsman RL Mollema ED Oort FJ Hoos AM de Haes JC Using standardized video cases for assessment of medical communication skills - Reliability of an objective structured video examination by computer Patient Education and Counseling 2006 60 1 24 31 10.1016/j.pec.2004.11.010 16332467

[B31] Mazor KM Haley HL Sullivan K Quirk ME The Video-based Test of Communication Skills: Description, development, and preliminary findings Teaching and Learning in Medicine 2007 19 2 162 167 10.1080/10401330701333357 17564544

[B32] Wong FKY Kember D Chung LYF Yan L Assessing the Level of Student Reflection from Reflective Journals J Adv Nurs 1995 22 1 48 57 10.1046/j.1365-2648.1995.22010048.x 7560535

[B33] Kember D Jones A Loke A Determining the level of reflective thinking from students' written journals using a coding scheme based on the work of Mezirow International Journal of Lifelong Education 1999 18 1 18 30 10.1080/026013799293928

[B34] Duke S Appleton J The use of reflection in a palliative care programme: a quantitative study of the development of reflective skills over an academic year J Adv Nurs 2000 32 6 1557 1568 10.1046/j.1365-2648.2000.01604.x 11136426

[B35] Wald HS Reis SP Borkan JM Reflection rubric development: evaluating medical students' reflective writing Medical Education 2009 43 11 1110 1111 19799722 10.1111/j.1365-2923.2009.03470.x

[B36] Devlin MJ Mutnick A Balmer D Richards BF Clerkship-based reflective writing: a rubric for feedback Medical Education 2010 44 11 1143 1144 20946509 10.1111/j.1365-2923.2010.03815.x

[B37] Ward JR McCotter SS Reflection as a visible outcome for preservice teachers Teach Teach Educ 2004 20 3 243 257 10.1016/j.tate.2004.02.004

[B38] Andrade HG Using rubrics to promote thinking and learning Educ Leadersh 2000 57 5 13 18

[B39] Popham WJ What's wrong–and what's right–with rubrics Educ Leadersh 1997 55 2 72 75

[B40] Moon JA Reflection in learning and professional development: theory and practice 1999 Kogan Page, London

[B41] Bourner T Assessing reflective learning Educ Train 2003 45 5 267 272 10.1108/00400910310484321

[B42] Hayes AF Krippendorff K Answering the Call for a Standard Reliability Measure for Coding Data Communication Methods and Measures 2007 1 1 77 89 10.1080/19312450709336664

[B43] Crossley J Davies H Humphris G Jolly B Generalisability: a key to unlock professional assessment Medical Education 2002 36 10 972 978 10.1046/j.1365-2923.2002.01320.x 12390466

[B44] Mezirow J and Associates Learning as Transformation: Critical Perspectives on a Theory in Progress 2000 Jossey-Bass, San Francisco

[B45] Eva KW Regehr G Self-assessment in the health professions: A reformulation and research agenda Academic Medicine 2005 80 10 S46 S54 16199457 10.1097/00001888-200510001-00015

[B46] Makoul G Zick AB Aakhus M Neely KJ Roemer PE Using an online forum to encourage reflection about difficult conversations in medicine Patient Education and Counseling 2010 79 1 83 86 10.1016/j.pec.2009.07.027 19717269

[B47] Dahlgren LO Eriksson BE Gyllenhammar H Korkeila M Saaf-Rothoff A Wernerson A Seeberger A To be and to have a critical friend in medical teaching Medical Education 2006 40 1 72 78 10.1111/j.1365-2929.2005.02349.x 16441326

[B48] Sargeant J Eva KW Armson H Chesluk B Dornan T Holmboe E Lockyer JM Loney E Mann KV van der Vleuten CPM Features of assessment learners use to make informed self-assessments of clinical performance Medical Education 2011 45 6 636 647 10.1111/j.1365-2923.2010.03888.x 21564201

[B49] Mushquash C O'Connor BP SPSS and SAS programs for generalizability theory analyses Behavior Research Methods 2006 38 3 542 547 10.3758/BF03192810 17186766

[B50] Kreiter CD Bergus GR Case specificity: Empirical phenomenon or measurement artifact? Teaching and Learning in Medicine 2007 19 4 378 381 10.1080/10401330701542776 17935468

